# Application of Multitemporal Change Detection in Radar Satellite Imagery Using REACTIV-Based Method for Geospatial Intelligence

**DOI:** 10.3390/s23104922

**Published:** 2023-05-19

**Authors:** Jakub Slesinski, Damian Wierzbicki, Michal Kedzierski

**Affiliations:** Department of Imagery Intelligence, Faculty of Civil Engineering and Geodesy, Military University of Technology, 00-908 Warsaw, Poland

**Keywords:** SAR, change detection, time series, multitemporal analysis, REACTIV, coefficient of variation, geospatial intelligence

## Abstract

Constant monitoring of airports and aviation bases has become one of the priorities in today’s strategic security. It results in the necessity to develop the potential of satellite Earth observation systems and to intensify the efforts to develop the technologies of processing SAR data, in particular in the aspect of detecting changes. The aim of this work is to develop a new algorithm based on the modified core REACTIV in the multitemporal detection of changes in radar satellite imagery. For the purposes of the research works, the new algorithm implemented in the Google Earth Engine environment has been transformed so that it would meet the requirements posed by imagery intelligence. The assessment of the potential of the developed methodology was performed based on the analysis of the three main aspects of change detection: analysis of infrastructural changes, analysis of military activity, and impact effect evaluation. The proposed methodology enables automated detection of changes in multitemporal series of radar imagery. Apart from merely detecting the changes, the method also allows for the expansion of the change analysis result by adding another dimension: the determination of the time of the change.

## 1. Introduction

As a result of significant technological progress, access to sources of intelligence data have become more common. This results in the overlapping of certain types of intelligence. The geospatial intelligence (GEOINT) is a synergy of the imagery intelligence (IMINT) and geospatial data (GEOINFO) [1]. It includes comprehensive collection and analysis of data about the geographical space and the enemy with use of various intelligence techniques and methods (mainly based on the possibilities offered by imagery intelligence) in order to prepare multi-layered geospatial intelligence information. Thus, GEOINT is a strategic branch of intelligence, which may often decide the success or defeat of a country confronted with a crisis or even a war. Continuous monitoring of the areas of interest and detecting the occurring changes is the main task of satellite imagery intelligence. It enables the detection of threats and updates the state of knowledge about the objects that might potentially pose such risks [2].

Due to described IMINT needs, constant, autonomous access to data from satellite observations of the Earth is necessary, regardless of the time of day and the atmospheric conditions. Although radar imagery does not provide imagery that would be so easy to interpret as images from optic sensors, these images are perfectly suitable for comprehensive scanning of large areas and detecting the changes that emerge there. The main advantage of observations with the use of radar sensors is the possibility of constant monitoring.

As opposed to optical instruments, radars with synthetised aperture may conduct observations regardless of the current weather of lighting conditions in the given part of the globe.

IMINT is also closely related to the concept of situational awareness. Situational awareness (SA) is the ability to recognise objects in the environment, comprehend their significance, and predict how they will behave in the near future. IMINT and GEOINT systems offer a way to assign geographic aspects to data. Complex dynamics, patterns, and relationships can be shown, examined, and comprehended in a totally new way within this visual framework. This raises the concept of “situational awareness” to a completely new level and makes geographic analysis possible, which is a completely new and potent sort of capability [3,4].

The constantly growing dynamics of change in the security area results in the need to at least partly automate the intelligence processes. Currently, most IMINT′s studies are still based on manual work and the experience of analysts. Therefore, there is a need to develop methods to improve the work of analysts, especially in the field of change detection. Automated systems may perform numerous analyses independently, thus releasing humans from the need to make physical and partly intellectual efforts. Moreover, they usually perform the analyses faster and more precisely than humans. Long-time series analysis brings potential information on land cover changes while also improving the quality and accuracy of change information generated from remote sensing. Due to the rapid development of SAR satellite remote sensing technology, a significant number of multitemporal pictures are easily available, which drives change detection from bitemporal to dense long-time series analysis. A major study has focused on using SAR image time series (ITS) to rapidly estimate the precise change time and detect the change patterns.

At present, change detection methods based on time series analysis mainly focus on acquiring single change information, e.g., spatial information [5] and temporal information. Although there are novel visualization models, such as REACTIV algorithm, simultaneously incorporating change frequency and change time, it is still lacking a description of change patterns. A novel unified framework for long-time series SAR image change detection and change pattern analysis (SAR-TSCC) was proposed for land cover change mapping [6].

The research works were based on the three main aspects of IMINT change detection: analysis of infrastructural changes, analysis of military activity, and Battle Damage Assessment (BDA), which were performed on military objects. The conducted research confirmed the effectiveness of the use of radar data and Rapid and Easy Change detection in radar Time-series by Variation coefficient (REACTIV) algorithm in imagery intelligence.

### 1.1. Related Works—Change Detection

The detection of changes is the process of detecting and identifying the differences between two or more images captured at different times [4]. The main problem here is the selection of pixels that differ significantly between the images. Such significant changes may be, for example, the occurrence or disappearance of objects, the movement of objects in relation to the background, or changes in the shapes, brightness, or colours of objects [5].

The factor that hinders the detection of changes in digital imagery is the presence of certain errors and distortions that are caused, among others, by sensor errors, the influence of the representation method, the angle of imaging, or atmospheric influences. The literature provides multiple methods of change detection and several approaches to their classification [5]. Such multiplicity results from the large number of areas where change detection is applied, from agriculture, through military intelligence to medical diagnostic procedures. The selection of the approach in detecting changes depends on multiple aspects: the type of available data, the expected resulting products, or how quickly we will need the results of such analyses [7]. Change detection is a process that depends on certain analytical questions. Its starting and end points may vary. For example, identifying the areas where a change has occurred may be an initial stage before checking the scope of these changes. In some analyses, the aim of change detection may be to determine whether and where changes have occurred. Other analyses may focus on the degree of changes in the given area, while still other research may focus on the question: “what has the area changed into?”

Zhao [5] presented a classification of types of changes according to the change in land cover and the cause of the change. Changes in land cover may be divided into:

- Change from one class of land cover into another;
- Change of shape: spreading, shrinkage, or transformation;
- Change of position;
- Division or merging of neighbouring areas.

Changes may also be classified according to their cause into the following groups:

- Short-term change;
- Repeatable (seasonal) change;
- Directional change (e.g., urban development);
- Multidirectional change (e.g., simultaneous deforestation and reclamation);
- Sudden events (e.g., a natural disaster).

The most commonly used approach in detecting changes in digital images is so-called binary change detection, sometimes also referred to as bitemporal or two-dimensional. It consists of detecting the changes between two images. There are tens of algorithms to detect changes. Ilsever and Ünsalan [4] divided the two-dimensional change detection methods into four basic groups: Pixel-Based CD, Transformation-Based CD, Texture Analysis Based CD, and Structure-Based CD. The fifth category includes other techniques that are a combination of the previously mentioned methods.

Pixel-based change detection techniques are successfully applied in the analysis of medium- and low-resolution SAR imagery, but very high-resolution images that are becoming increasingly common may pose a challenge due to large changes in the intensity within objects and elements of land cover [8].

Three steps are often used in traditional SAR image change detection techniques: (1) image pre-processing, (2) differential image generation, and (3) differential image analysis to pinpoint the changed area [9]. The spatial correspondence between bitemporal SAR images must be established during picture pre-processing using image registration [10], which can provide strong image consistency in the spatial domain [11]. Although the creation and analysis of DI (differential images) are the main components of SAR change detection, the quality of DI and the approach used to analyse DI will have a significant impact on the detection’s final outcomes [12]. Speckle effects multitemporal SAR image-based change detection. Dekker [13] has shown that speckle increases false and missed alarm rates when thresholding the SAR ratio image. Filtering the individual SAR images prior to comparison is the conventional method for removing the effect of speckles in SAR-based change detection [14]. It is challenging to create a good difference map because speckle noise, which is an unavoidable flaw in the SAR imaging process generated by the fading of the radar target echo signal, blurs image details and lowers image quality. Then, there are the issues with producing performant classifiers and good DI.

As for the methods of change detection that are characteristic of radar imagery, the literature [15,16] mentions two main approaches to this problem: amplitude and coherent change detection. Amplitude change detection, ACD, also referred to as the non-coherent method, is based on the analysis of changes in the distribution of the ratio of energy signal sent to the reflected energy. Due to certain similarities to panchromatic electro-optical imagery, changes in amplitude images may be detected with the use of similar methods [17]. The method of detecting non-coherent changes is effective in the evaluation of changes in large areas. Additionally, it is also characterised by high-efficiency in terms of time and costs.

Based on whether prior knowledge is necessary, there are two types of non-coherent change detection methods based on SAR images: supervised and unsupervised methods. The unsupervised change detection method is more frequently employed in practice [9,18] than the supervised methodology, which is labour- and time-intensive because it depends on genuine reference data [19].

The advantage of unsupervised change detection algorithms is that no prior knowledge of the research domain (training data) is necessary. A binary change map displaying changed vs. unchanged areas is typically the result of an unsupervised change detection algorithm. The fundamental drawback of these algorithms is the absence of comprehensive from–to change data [14]. The thresholding method [20] and the clustering method [21] are frequently employed in unsupervised change detection. The thresholding approach selects the optimal threshold, with which the pixels of DI are divided into change class and invariant class. It is set to white (change class) if it is larger than the threshold and black if it is less than or equal to the threshold (invariant class) [12].

Usually, supervised change detection is carried out using post-classification comparison logic. It consists of classifying each image in the multitemporal dataset independently using the same classification scheme. The detailed from–to change information can then be extracted by comparing the classified images on a pixel-by-pixel basis [22]. The main drawback of the supervised change detection method is the need for high-quality training data to classify each image in the multitemporal dataset. This turns out to be rather difficult to achieve, especially for older images [14].

The three most often used SAR image processing algorithms for creating change images are image ratio, log ratio, and likelihood ratio [23]. In terms of change detection algorithms, pixel-based and object-based approaches, and supervised and unsupervised change detection, are employed. When utilising automatic thresholding methods, unsupervised change detection has been very popular recently. These methods analyse the changing image (such as a ratio or differenced image) and produce a binary change map that contrasts classes that have changed and those that have not. Object-based analysis can greatly lessen the computing strain for high-resolution imagery [14]. When comparing object-based change detection to pixel-based methods, it was found to be less sensitive to picture co-registration errors [24].

In non-coherent change detection, methods based on the statistical analysis of SAR images are also important [15]. Hachicha and Chaabane [25] proposed two different types of change indicators that were developed based on the assumption that SAR amplitudes are Rayleigh distributed. While the first indicator, the Rayleigh ratio detector, operates per pixel and uses first-order statistics, the second indicator, the Rayleigh Kullback–Leibler divergence, uses higher-order statistics for the comparison [14].

As opposed to binary change detection, multitemporal change detection (MT CD) is used to detect changes between more than two images [5]. While bi-temporal change detection usually enables to show two main properties of changes, i.e., their direction and intensity, multitemporal methods allow to obtain other, additional properties, such as the type of change, its stability, and time of occurrence.

The classification of the types of change, according to Zhao [5], refers to whether the given change progresses gradually, with time (evolution) or whether it has the nature of an impulse that takes place at a specific time. Stability of change defines whether the change is a permanent, one-off event or whether it repeats multiple times, at the same location, at regular or irregular intervals. The time of change, which is particularly important for long temporal series, answers the question: “When did the change occur?” This results in a significantly higher information capacity for multitemporal series of images. On the one hand, it offers large possibilities for improving the efficiency of data use, but on the other hand, it may pose certain difficulties. Changes in multitemporal series have at least five properties, and, as a typical RGB image consists of three channels, it becomes impossible to visualise them all at the same time [8].

Multitemporal methods of change detection in radar imagery are not as widely discussed in the literature as the binary change detection (CD) method. In a wide range of applications, change detection using multitemporal remote sensing imaging is crucial. Examples include urbanization [26], deforestation [27], flooding [28], infrastructure monitoring [29], disaster monitoring, and damage assessment [30]. Reference [14], however, there are only a few publications on the use of SAR image change detection in the military and security—here, an example can be given Novak [16] or Canty [31].

The robustness of single bi-date change detection can be improved by using more images, and the temporal information can help distinguish between distinct classes of change and hence aid in the characterisation of changes [32]. Although the use of time series in this context is hugely interesting, there is less research on the detection of changes in the time series of N dates in the literature since access to sufficient data for a long time series is only recently available, notably for SAR images [33]. A vast majority of the existing methods of detecting changes in a series of SAR images may be divided into two following types [33,34]: simultaneous comparison (offline method) and comparison of pairs (online method). In the first approach, pixels from the whole multitemporal series are compared simultaneously in order to recognise where and when the change occurred and focuses on how to specify a test to determine whether a statistical population as a whole is homogeneous. Detecting a probable statistical rupture without attempting to date it precisely is possible if the hypothesis is rejected. Methods from the second group are used to process data as they are obtained so as to detect the change as soon as possible after it has occurred. This paper focuses on the first approach, for which several characteristic methods may be distinguished. The first one is the MIMOSA method (Method for generalised Means Ordered Series Analysis) [5,33], which is based on the statistical analysis of the function of probability density between two square and geometric means. A slightly different approach is represented by the Omnibus method, whose algorithm is based on performing a simultaneous test of the hypothesis of the homogeneity of distributions [34]. Another solution is the application of the coefficient of variation (expressed as the standard deviation × mean^−1^). It was precisely this approach that is implemented in the REACTIV method [33] was used in further research.

Using artificial intelligence AI, in particular, convolutional neural networks CNN to learn features from remote sensing images to study the changes in bitemporal images has become a common method in the field of change detection. Artificial intelligence (AI) techniques, often known as machine learning, can improve performance in a variety of data-processing jobs. It can be described as a system’s capacity to accurately understand outside input, to learn from that data, and to use that learning to accomplish certain objectives and tasks through adaptable change. Artificial intelligence (AI) techniques, often known as machine learning, can improve performance in a variety of data-processing jobs. It can be described as a system’s capacity to accurately understand outside input, to learn from that data, and to use that learning to accomplish certain objectives and tasks through adaptable change [12,35].

To increase the precision and automation of change detection, numerous novel methods, including AI techniques, have been developed. Numerous RS studies have revealed that in terms of feature extraction, AI-based change detection systems are superior to conventional methods [36,37]. AI algorithms can represent the relationship between the image item and its real-world geographical feature as accurately as possible because of their excellent modelling and learning capabilities, which enables the identification of more accurate change information. Data acquisition is the initial phase in both the classic change detection flow and the AI-based change detection flow, and the goal of change detection is to obtain the change detection map for diverse applications. While AI-based systems normally require an additional training set creation process and an AI model training process for change detection, traditional approaches typically involve two phases after data preparation, including a homogenisation process and a change detection process [38].

To sum up, there is no one solution that can solve all change detection issues due to their complexity. Different applications demand different strategies, and various forms of remotely sensed data necessitate taking into account sensor-specific concerns [9].

### 1.2. Research Purpose

A new field of Earth observation has emerged: multitemporal remote sensing. The remote sensing community is faced with significant obstacles and numerous opportunities due to the growing number of Earth Observation systems with improved capacity and free access to terabytes and petabytes of multitemporal data with worldwide coverage. In order to process longer and denser time series data, novel processing and data mining techniques are required. Additionally, efficient change detection techniques must be developed in order to process SAR images; in particular, change detection methods need to be advanced to meet modern requirements [14].

In order to use large SAR image stacks for IMINT and support situational awareness, it is necessary to change a large volume of data into a format that allows it to be processed and interpreted as information. The previous chapter found and discussed a number of existing change detection and visualisation techniques, but only one of them was able to represent the time of change in the final difference image. This developed method was chosen to visualise image stacks in this research.

The main research problem for this thesis was: How can large SAR image time series be used efficiently in Imagery Intelligence and supporting situational awareness? This problem was approached by synthesising the image stack to a single image using a new algorithm based on a modified REACTIV core, which shows changes throughout the image stack, which would show the changes between different dates and enable extracting more information from original images. In order to address this issue, two research questions were established:

- How to use a single image to show changes across long SAR image time series?
- How can visualisation be effectively used in the intelligence process and support situational awareness? (In particular with regard to reconnaissance of air bases infrastructure and equipment).

The article presents a method of detecting changes in SAR image series for the purposes of IMINT. A new algorithm based on the REACTIV core was presented, and its performance was tested using the Google Earth Engine platform. The modification included adjustment of difference image display parameters (gamma correction) and data fusion with optical imagery to allow recognition of features easier and evaluate the changes in the context of surroundings (with adjustment of opacity between radar and optical images). The effectiveness of the proposed image processing method was verified on the basis of a comparative analysis with reference optical and SAR image data.

## 2. Materials and Methods

Rapid and Easy Change detection in radar Time-series by Variation coefficient (REACTIV) is a highly efficient algorithm for the detection and visualisation of changes in time series. It was developed in a project by the team of the French research centre ONERA (Office National d’Etudes et de Recherches Aérospatiales), Palaiseau, France [39]. Detecting the change is important—it is the main aim of the method, but the knowledge about when it occurred is useful as well.

The developed algorithm enables the visualisation of the detected changes along with the information about the date of the change. However, if multiple changes have occurred in the same area (e.g., an object that regularly appears and disappears), it will not be possible to present all the information in a single three-channel image, so in such an event, the REACTIV visualises the colour that corresponds to the date of the most intensive activity in the whole temporal series [40].

REACTIV operates based on a multitemporal series of radar images. In this case, such a time series may be defined as a four-dimensional set of data in the form of the quantifier:

(1) $$\forall_{i}\in \;⟦1\ldots w⟧,j\in ⟦1\ldots h⟧,l\in \left\{1;2\right\},\;co\;daje\;p_{ij}\left\{p_{ijl}^{\left(1\right)},p_{ijl}^{\left(2\right)},\ldots ,p_{ijl}^{\left(T\right)}\right\},$$

where: $\forall_{i}$—quantifier (‘for all *i*’), *w*—spatial elements on the x axis, *h*—spatial elements on the y axis, *T*—elements on the temporal axis, and *p_ijl_*—temporal pixel. The x and y axes are spatial axes. The z axis corresponds to polarisation (number of available polarisation configurations, from 1 to 4). Each temporal pixel, denoted as $p_{ijl}^{\left(T\right)}$, is defined by pixel coordinates (*i*, *j*) on the x and y axes, the selected polarisation of pixel *l*, and the time parameter (*T*). Thus, a time series of images of the size of 500 × 500 pixels, consisting of 100 images (100 dates), gives a four-dimensional data object of the shape (500, 500, 2, 100), where 2 is the polarisation dimension [40].

**Figure 1:**
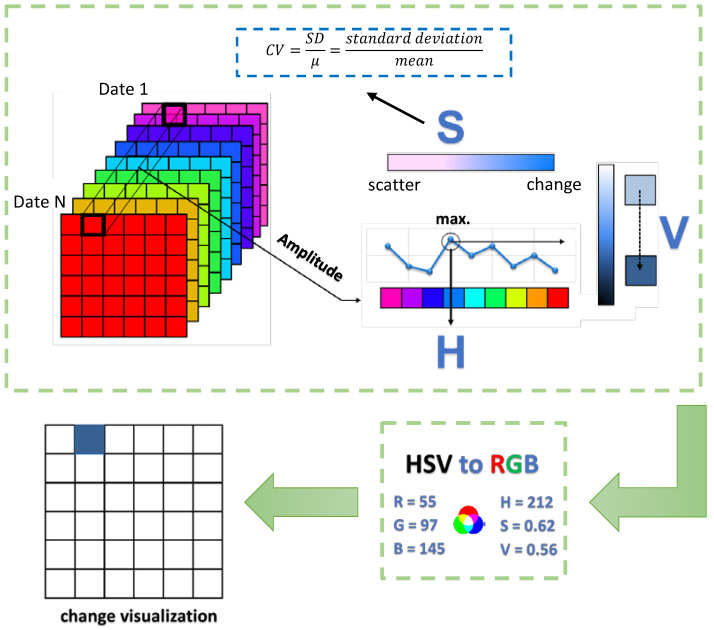
Workflow for REACTIV multitemporal change detection.

In the REACTIV method, the hue represents the time, saturation represents change, and the value corresponds to the intensity of reverse refraction. The algorithm searches for the maximum intensities of each pixel in the whole time series and associates this intensity with the value component and the corresponding date with the hue channel. The operation of the algorithm is explained in Figure 1.

### 2.1. A Hue Component: The Temporal Dimension

**Figure 2:**
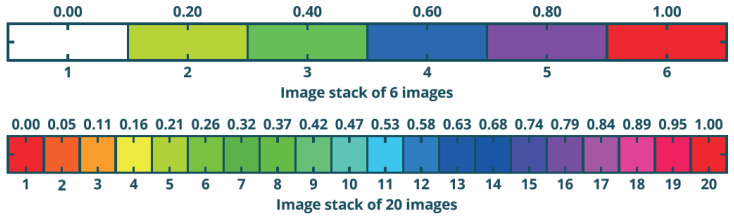
Correlation between hue and number of images in the stack—based on [8].

The hue represents the colour from the HSV (Hue, Saturation, and Value) colour circle that is used. This component encodes the information about the date of the detected change. The original scope of the hue in the HSV space is defined as a number from the range from 0 to 360°, although in this case, it is normalised and takes the value from 0 to 1. The REACTIV method divides the scope by the number of images in such a way that the first image has a value of 0 and the last one of 1. This means that the number of hues depends on the number of images in the set, which is presented in Figure 2.

The hue values are distributed proportionally to the axis of the temporal series, which means that images captured at short time intervals are clustered in similar hues, while images obtained at longer intervals are represented by significantly different colours.

**Figure 3:**
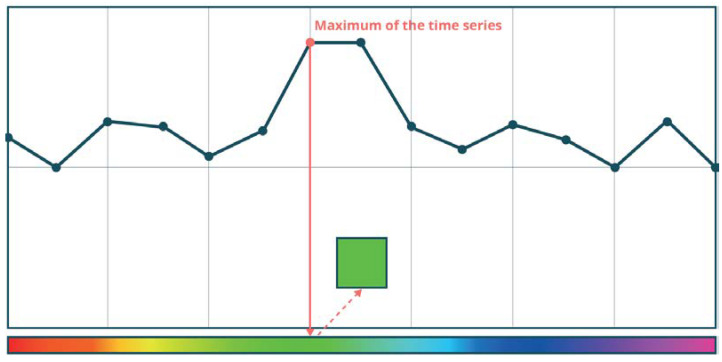
Hue determination in the REACTIV method—based on [40].

The hue is determined based on the selection of the temporal pixel $p_{ijl}^{\left(T\right)}$, of the maximum intensity in the time series. Thus, when a change occurs, it is possible to determine approximately when it occurred in the observed period. This may be calculated from the formula:

(2) $$H_{i}=\frac{t_{i}-t_{1}}{t_{n}-t_{1}},$$

where: *t_i_* is the date for which the hue value is calculated, *t*_1_ is the first image in the series, and *t_n_* is the last image in the series. The principle of hue selection is explained in Figure 3, where the maximum intensity of a multitemporal series in the given point fell to the green colour, and this is the hue in which the change would be visualised in this case. Such matching is particularly beneficial in the event of single changes in the image, such as an object (e.g., a vessel) appearing. In the cases of permanent changes, i.e., the appearance or disappearance, the colour will represent one of the dates when the intensity of negative refraction was particularly high, so it will most likely represent the date when the object appeared or disappeared.

### 2.2. Saturation Component: Change in Intensity

Saturation is responsible for the intensity of the colour defined by the previously selected hue. This parameter is linked to the intensity of change: the more significant the change, the more saturated the colour will be, and the more saturated the colour, the more vivid it appears. On the other hand, less saturated tones seem muted and closer to grey. This means that the “lighter” points in the map represent places where small changes occurred in time [33].

In order to determine the saturation component, REACTIV uses the coefficient of variability (*CV*) as the main parameter that represents change. Pixels with a low variation of coefficient will have low saturation and vice versa. Due to that, even if the given pixel is linked to a specific date by its hue (by the hue component), it may be displayed in grayscale when the coefficient of variability is low. Pixels with a high *CV* will have highly saturated colours, and thus they will be visualised as change [39].

The amplitude of speckle distribution in SAR images is consistent with the Rayleigh-Nakagami model. In the REACTIV method, it is assumed that the temporal profile of a pixel in a multitemporal series of images is also consistent with this model [8]. As a result, the coefficient of variability may be defined as follows:

(3) $$CV=\frac{SD}{\mu },$$

where *SD* is the standard deviation and *μ* is the average value of the given pixel in the series. Using the Rayleigh-Nakagami model, the coefficient of variability may be determined as:

(4) $$CV=\sqrt{\frac{\Gamma \left(L\right)\Gamma \left(L=1\right)}{\Gamma {\left(L+1/2\right)}^{2}}-1},$$

where: *Γ* is a function of the gamma distribution and *L* is the number of “looks” (i.e., the samples used for averaging), which was used to filter the noise in the images.

In the event of clearly noticeable changes, the *CV* will be higher, due to a higher standard deviation. The coefficient of variability is normalised:

(5) $$CV\leftarrow \frac{CV-\mu }{10\;SD}+0.25$$

where: *SD* and *μ* are theoretical values for “stable” speckling. Normalisation reduces the level of saturation around stable zones and increases the range of the area identified as change [8].

### 2.3. Value Component: Intensity of SAR Signal

**Figure 4:**
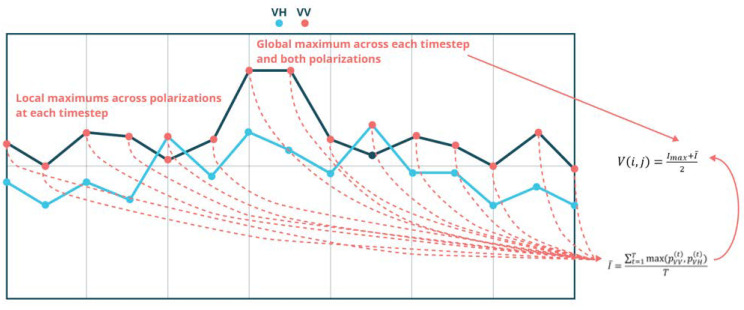
Scheme for calculating the component value in the REACTIV method based on [16].

The last parameter that is necessary for the visualisation of changes is the value component, which determines the final appearance of the change detection map. This value falls into the range from 0 to 1 (from dark to light, in reference to the colour determined by the hue value). The procedure of the determination of the value component is explained in Figure 4. This component is calculated based on the maximum signal values in both polarisations, but its value may be adjusted empirically with use of a non-dimensional coefficient λ (so as to achieve a clearly legible product). In this case, the intensity of the value of pixel *p_ij_* was calculated on the average sum of the overall maximum intensity *I_max_* and the average of local maximum intensities $\bar{I}$ [15].

(6) $$V\left(i,j\right)=\frac{I_{max}+\bar{I}}{2},$$

### 2.4. Sentinel-1 Data

Sentinel-1 is the first mission of the European Space Agency (ESA) that was prepared as part of the Copernicus programme. The aim of the mission is to ensure long-term coverage of the whole globe with radar data. This includes land, sea zones, European coastal zones, and oceans. The interval for recording images for the same satellite scene and a single satellite is 12 days (above the equator), while for two Sentinel-1 satellites, it ranges from 2 to 6 days (depending on the latitude). The specifications of the Sentinel-1 constellation are presented in Table 1.

The conducted analyses were based on data obtained in the Interferometric Wide Swath (IW) mode that records images in a belt of the width of 250 km with a spatial resolution 5 × 20–30 m for a single impulse in the so-called TOPSAR mode. The Sentinel-1 scene in this mode consists of three belts, and each of them is additionally divided into eight or nine fragments. This is the basic imaging mode of this system [17].

### 2.5. GEE Platform

Google Earth Engine (GEE) is a platform that operates in the computational cloud, which is designated mainly for analysing large sets of remote sensing and vector data. The GEE servers store vast collections of publicly available data from satellite observations of the Earth that were recorded during the last 40 years in their archives. GEE is an environment that integrates the programming environment based on the JavaScript language with a large data repository (which includes a set of satellite data, vector data, GIS, socio-economic, demographic, meteorological and climatic data, and elevation models of the Earth [19,41].

The main module of the GEE is the code editor that constitutes an integrated programming environment IDE which operates in the Internet browser and is used for prototyping, writing scripts, and creating visualisations of complex spatial analysis with the use of the JavaScript language. The API interface provides access to function libraries developed for the purposes of GEE. They may be used to analyse data available in the cloud but also the user’s own geodata [3].

### 2.6. Test Area

**Figure 5:**
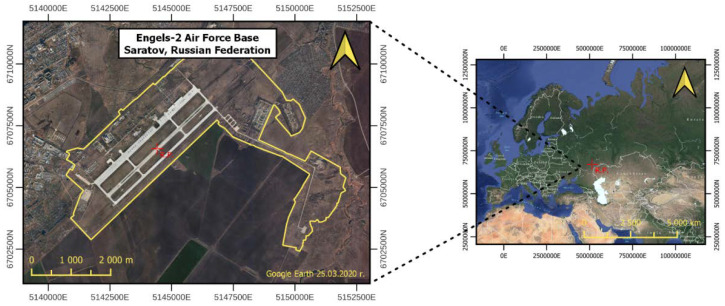
Localisation of study area: Engels-2 Air Force Base.

In order to employ this method for change detection in the military approach, certain modifications were necessary. Although the operating principle of the algorithm remained unchanged, certain transformations were introduced in order to improve and adjust the manner of displaying changes. The selected test object was the military strategic air force base Engels-2 located near the city of Saratov in the Russian Federation (Figure 5).

### 2.7. The Proposed Methodology

The aim of the authors of this method was to analyse changes in the natural environment, urban development, or the analysis of the formation of maritime routes based on the traffic of vessels.

**Figure 6:**
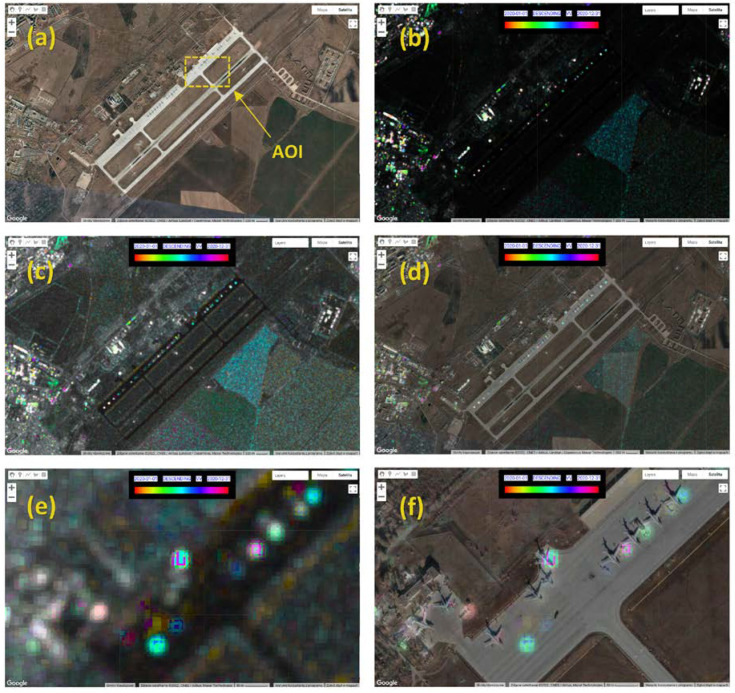
Results of change detection based on the example of Engels-2 air force base: (**a**) high-resolution image in the visible spectrum, with the marked area of interest AOI, (**b**) application of the REACTIV algorithm in the original form, (**c**) results after the application of gamma correction, (**d**) results after the application of data fusion, (**e**) results after the application of gamma correction for AOI, (**f**) results after the application of data fusion for AOI.

Change detection was conducted in GEE on a series of multitemporal images Sentinel-1 from the period from January to December 2020. The results of change detection and the proposed methods of modification are presented in Figure 6. Although the algorithm detected changes, their visualisation proved to be quite illegible (Figure 6b). This may have been caused by the relatively low resolution of the data used and the small surface of the analysed area. The raw captured signal can be enhanced by applying basic intensity transformation procedures, such as gamma correction. If the raw SAR image histogram is compressed within a narrow range of values (as in the case of the Sentinel-1 maps in GEE), this enables the expansion of the pixels intensity range and to reduce the speckle noise intensity in the data. In order to improve the interpretability of the results, the author proposed to apply gamma correction, which resulted in a significant improvement of the radiometrics, so that the results became clearer (Figure 6c,e). The second proposed modification consisted of the application of simple data fusion. The map of changes obtained with the use of the modified REACTIV algorithm was transformed by setting its transparency value to 0.5 (value adjusted experimentally), while at the same time, a background was added in the form of a high-resolution image in the visible spectrum (Google Satellite). As a result, the identification of the detected changes in reference to their position became significantly easier (Figure 6d,f). Source code of the source code of the analysed methodology is available on GitHub (URL: https://github.com/jakubslesinski/REACTIV_JS) (accessed on 10 May 2023).

## 3. Results

**Figure 7:**
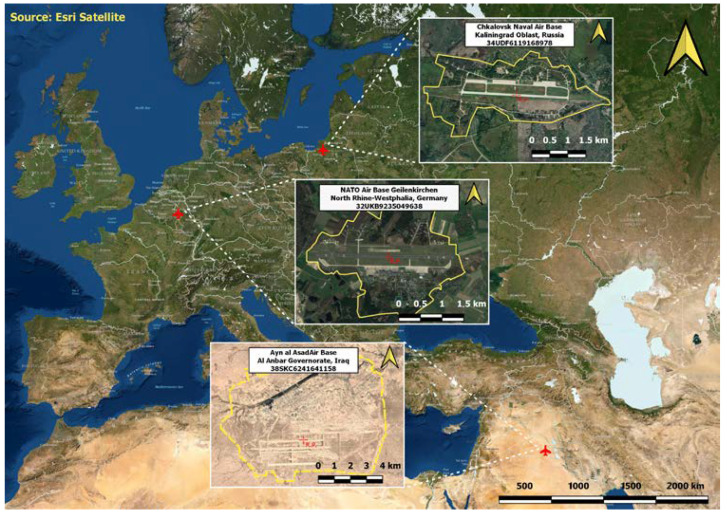
Location of the areas for which the methodology has been tested.

In order to evaluate the potential of the script developed in the GEE for imagery intelligence, the further stages of the research works consisted in conducting analyses based on the three main aspects of change detection: analysis of infrastructural changes, analysis of military activity, and Battle Damage Assessment (BDA). All the analyses were performed on military objects (Figure 7) based on open data.

### 3.1. Application of the REACTIV Method in the Detection of Changes in the Infrastructure of the Airport in Chkalovsk

The air force base of the Baltic Fleet of the Russian Federation Navy is located in the Kaliningrad Oblast, 10 km to the northwest of the city centre. The 689th Pokryshkin Guards Fighter Aviation Regiment, which was recreated in 2018, is stationed there. It is part of the 34th Composite Aviation Division of the Baltic Fleet. In recent years, the air force base in Chkalovsk has been thoroughly modernised. The runways, taxiways, and apron have received new concrete surface; the airport was also equipped with new electronic navigation systems. The fuel storage area (Petroleum, Oils, and Lubricants, POL) was also reconstructed, and a railway line connection was built [20]. The object selected for the detection of changes in the airport was the POL storage area.

**Figure 8:**
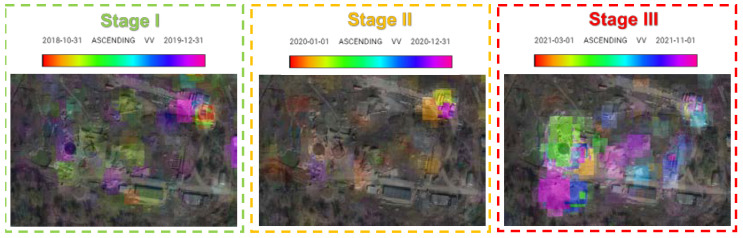
REACTIV change detection for individual stages.

After the analysis of the modernisation of POL in the years 2018–2021 on Google Earth images, the process was divided into three stages: preparation of the area (until 2020), transition period (2020), and proper modernisation (2021). The analysis of the results of the application of the REACTIV algorithm for specific stages (Figure 8) revealed an intensification of the modernisation works during stage III.

**Figure 9:**
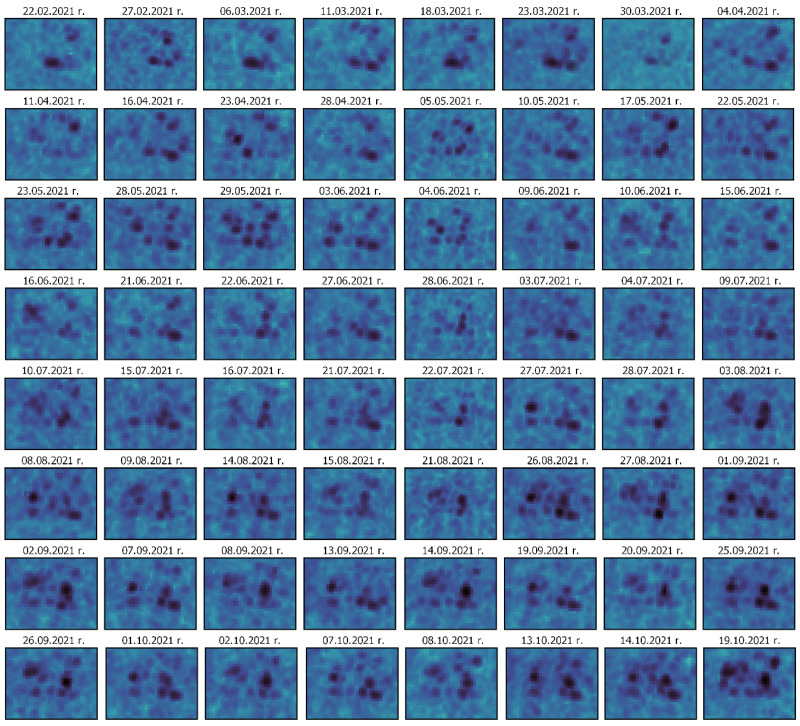
Sentinel-1 images time series of Chkalovsk AOI.

**Figure 10:**
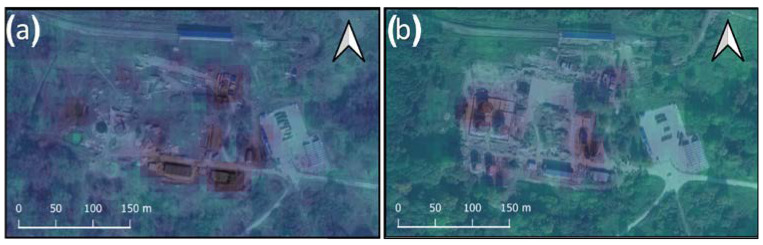
(**a**) Satellite image from Google Earth Pro (taken on 26 March 2021), with overlapped Sentinel-1 (taken on 23 March 2021), (**b**) Satellite image from Google Earth Pro (taken on 9 October 2021), with overlapped Sentinel-1 (taken on 14 October 2021).

In order to verify the changes detected with the use of the REACTIV algorithm and to assess the correctness of the determination of the date of the change by the algorithm, the illustration below (Figure 9) presents a series of the available images from Sentinel-1 (in the VV polarisation) from the period from February to October 2021. This confirmed the installation of the main fuel tanks in the second and third quarters of the year. Figure 10. shows a comparison of the first and last images from Figure 9. with the electro-optical data from Google Earth Pro.

**Figure 11:**
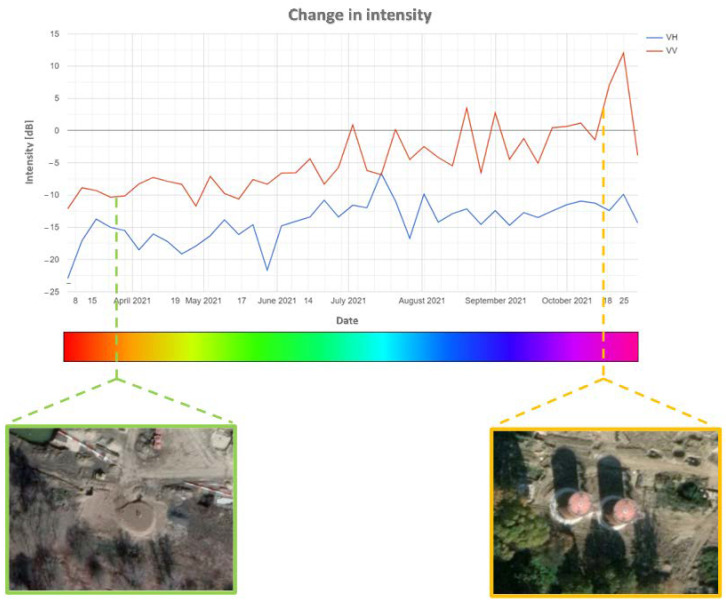
Graph of intensity changes in the southern part of POL storage, with confirmatory imagery obtained from Google Earth Pro.

The comparison of the diagram of the intensity of change (polarisation VV and VH) in time with high-resolution images from Google Earth Pro (Figure 11) also confirmed the correctness of both the change detection itself and the classification of the time of occurrence of change. This proves that the developed methodology is effective in detecting changes in airport infrastructure.

### 3.2. Application of the REACTIV Method in Monitoring the Activity of Equipment, Based on the Example of the NATO Air Force Base in Geilenkirchen

The Air Force Base in Geilenkirchen is situated in western Germany, near the Dutch border. A fleet of 14 AWACS (Airborne Early Warning And Control) aircraft are permanently stationed at the base as part of the NAPMO (NATO Early Warning and Control Programme Management Organisation) programme [42].

**Figure 12:**
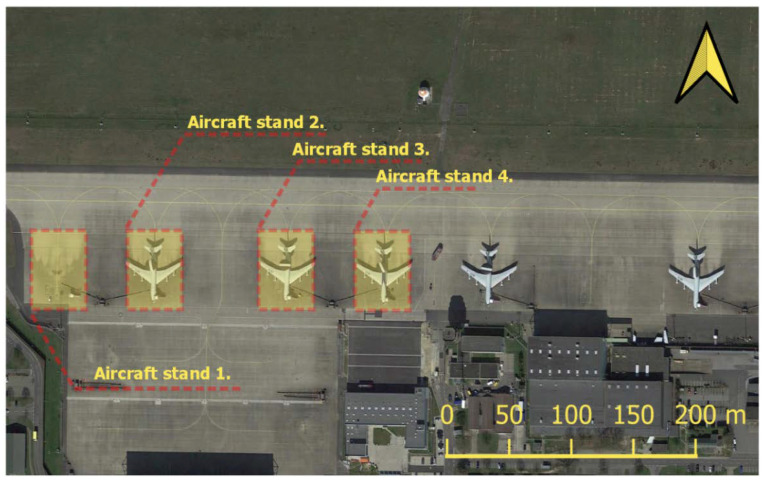
Locations of the Geilenkirchen AOIs (aircraft stands).

In order to analyse the military activity at the base, four aircraft stand for AWACS E3 Sentry were distinguished in the area of interest AOI. Their range and order are presented in Figure 12.

**Figure 13:**
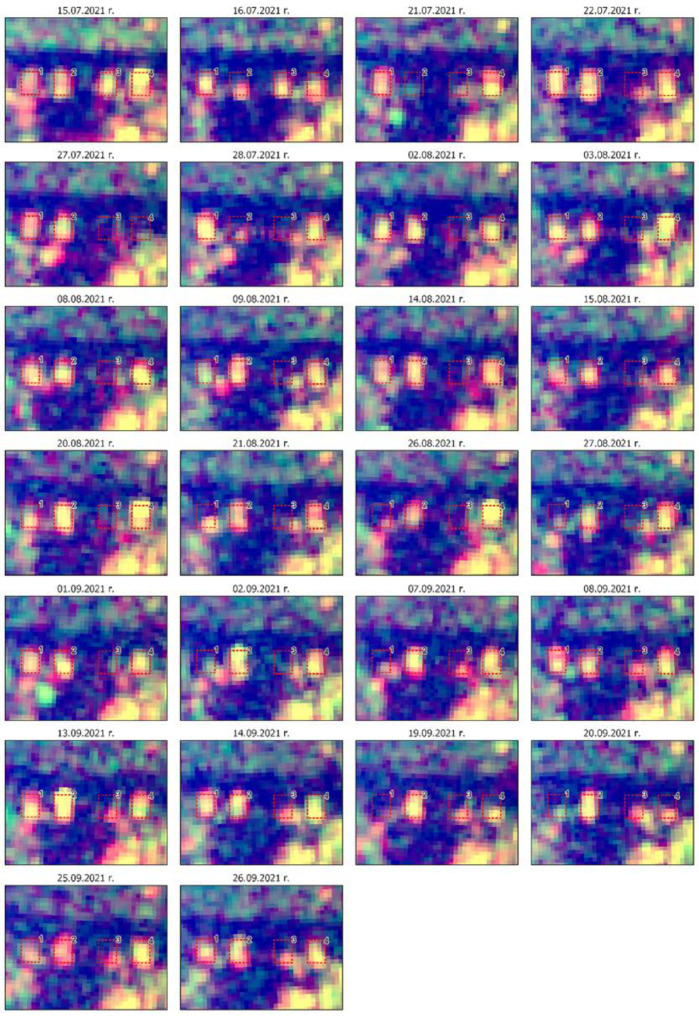
Sentinel-1 images time series of Geilenkirchen AOI (red boxes) with aircraft stands 1–4.

**Figure 14:**
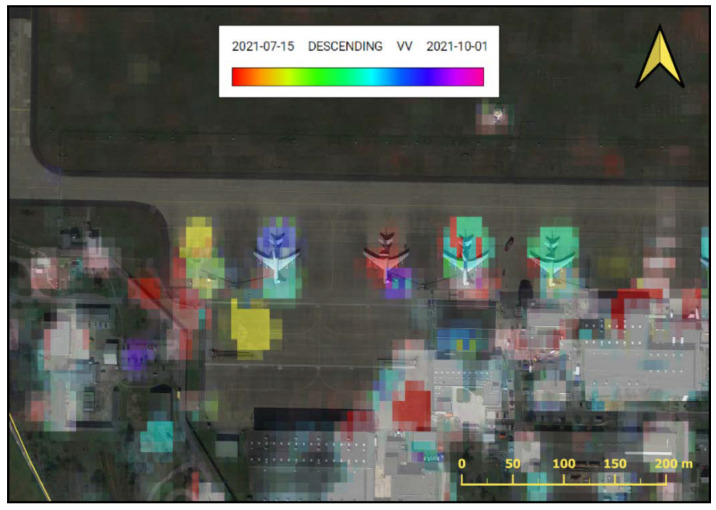
Change detection of each Geilenkirchen AOI.

The period selected for the analysis lasted from June to October 2021. This was the time of the most intensive exercises conducted by the Military Forces of the Russian Federation, including the large-scale exercise Zapad 2021. In order to present the activity of aircraft during that period, a multitemporal series of radar images was prepared for the Area of Interest (Figure 12). Pseudo colouring was applied with the use of a coloured composition consisting of images in the VV and VH polarisations. In Figure 13, one may notice frequent, rapid changes in the radar signal on the aircraft stands, which confirms the significant activity of the stationed aircraft during that time. The application of the REACTIV algorithm on this dataset resulted in the visualisation presented in Figure 14, which confirms the significant activity of the aircraft.

Here, the algorithm proved effective in detecting and localising changes. However, a disadvantage of the method may also be noticed. REACTIV determines the date of the occurrence of change based on the local maximum of the amplitude of the radar signal. If regular changes are analysed, consisting of the disappearance and reappearance of certain objects, the hue component will be calculated based on the change of the highest maximum intensity value.

**Figure 15:**
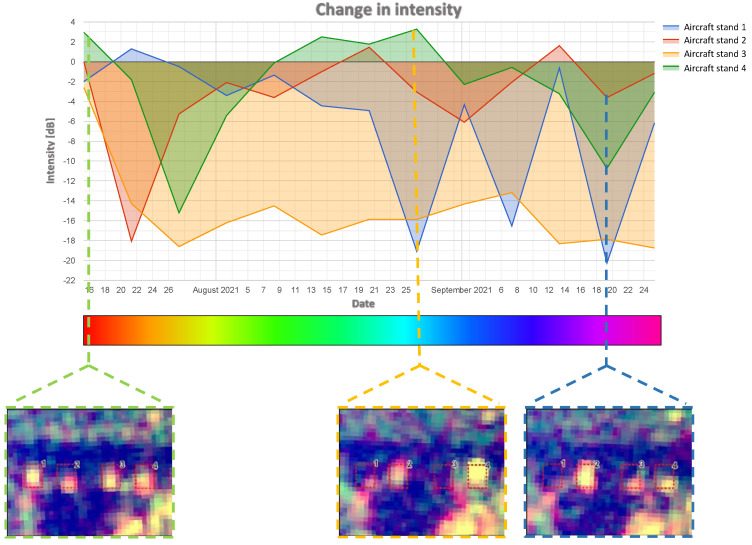
Graph of intensity changes in each aircraft stands (red boxes 1–4), with confirmatory Sentinel-1 imagery.

In order to reduce the influence of this disadvantage, a diagram of the changes in intensity in time may be used. Figure 15 presents such a diagram, which was generated in the GEE platform for all four analysed stands. Rapid decreases in intensity on the diagram may be interpreted as the disappearance of the object, while sudden increases represent its appearance. Below the diagram, radar images are presented that represent the analysed area at the given time.

The analysis of the activity in aircraft stand three (marked in orange on the diagram) reveals that the object (probably an aircraft) was present only at the beginning of the analysed period (high intensity of approx. 0 dB). In the later period, the intensity in this stand fluctuated around −16 dB, which may prove that the object disappeared permanently.

Intensified changes in the aircraft stands were observed in the first half of September, which may be connected with the activity of early warning aircraft related to the monitoring of the Zapad 2021 military exercise that took place on 10–16 September 2021. Thus, one may state that the application of the proposed methodology of detecting changes proved to be useful in monitoring the military activity of the airport, although with certain limitations (determination of the date for regular changes).

### 3.3. Application of the REACTIV Method in the Detection and Evaluation of the Effects of Impact on the Example of the Ayn al-Asad Base

The third analysed aspect of the application of the used change detection methodology in image intelligence is BDA (Battle Damage Assessment). The object of the analysis was the missile attack on the American part of the Ain al-Asad Base in Iraq that took place at night on 7–8 of January 2020. The Iranian attack on the American garrison of the US Air Force at the Ain al-Asad base was conducted with the use of over ten Fateh-313 ballistic missiles [43].

**Figure 16:**
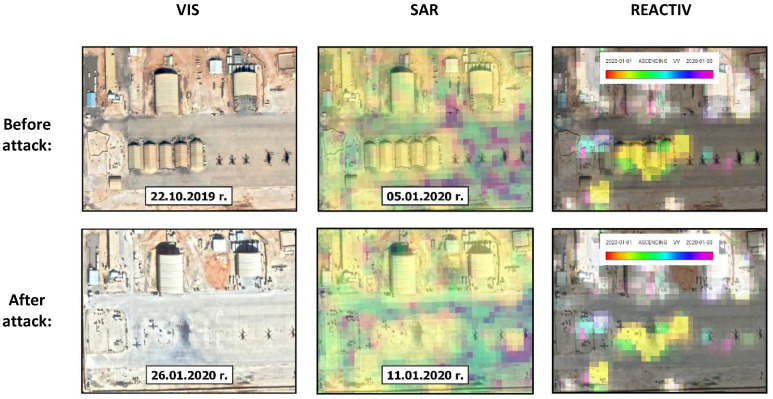
Comparison of images of the Ayn al-Assad Base before and after the attack, in RGB, SAR and REACTIV visualisation.

Knowing that the base was attacked on the night of the 7–8 of January, the period selected for analysis was January 2020. The results of change detection, along with the location of changes within the base, are presented in Figure 16. Although REACTIV failed to detect the superficial damages on the taxiway and the access roads to the hardened hangars (areas marked as 3 and 4 in Figure 16), the detection of the changes that resulted from the destruction of the building did not pose any problems.

Figure 16 presents a comparison of the images from the period preceding the attack and those that were obtained soon after it in reference to the area that suffered the most damage, i.e., the light hangars on the apron (area marked with 1). In spite of slight differences in the radiometrics between the radar images, the applied methodology enabled to locate of the changes, and the visualisation in yellow indicates that the changes occurred in the second week of January, which corresponds to the date of the attack.

### 3.4. Validation

Comparison of our methodology’s performance with other methodologies described above, such as the Omnibus test, requires the availability of trustworthy ground reference data. Such information is frequently difficult, if not impossible, to obtain. Due to the nature of the analysed objects (which are military bases), access to public high-resolution third-party geospatial data, which may constitute validation data, is extremely limited. Especially in the cases of Section 3.2 and Section 3.3, detailed verification would require a series of images with high temporal resolution. The largest amount of validation data could be obtained for the object from Section 3.1, which is why it was subjected to further quantitative analysis. Similar studies can now be carried out in a number of locations throughout the world thanks to the GEE platform. The discovered qualitative trends have not changed.

**Figure 17:**
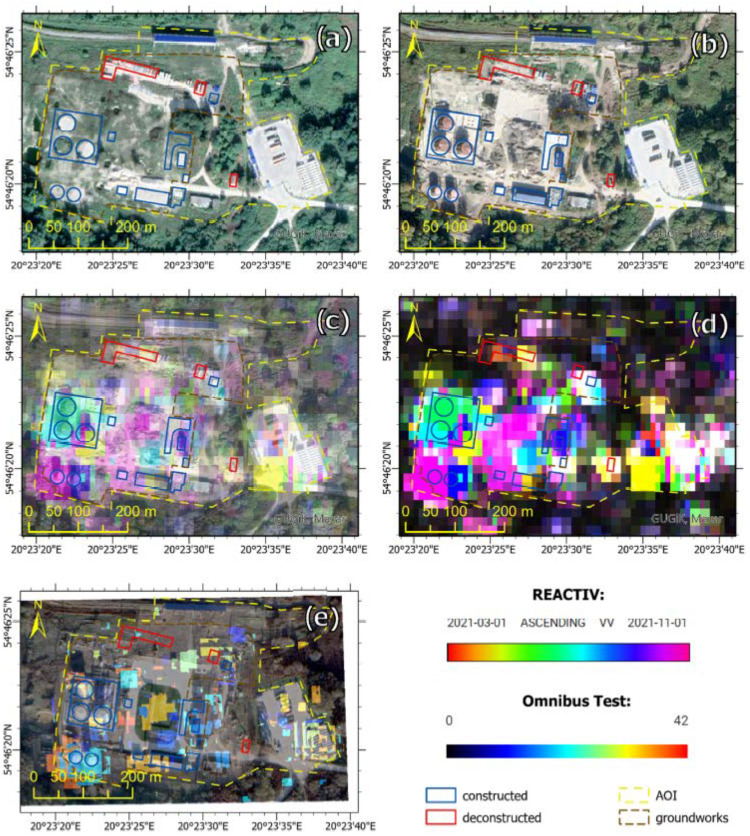
Comparison of changes detected in the POL storage area, in the Chkalovsk Air Base (Area 1): (**a**) Google Earth Pro electro-optical image from 26 March 2021, which detected changes, (**b**) Google Earth Pro electro-optical image from 9 October 2021, which detected changes, (**c**) Our algorithm change detection which background from 26 March 2021, (**d**) unmodified REACTIV change detection visualisation, (**e**) Omnibus test change detection visualisation.

**Figure 18:**
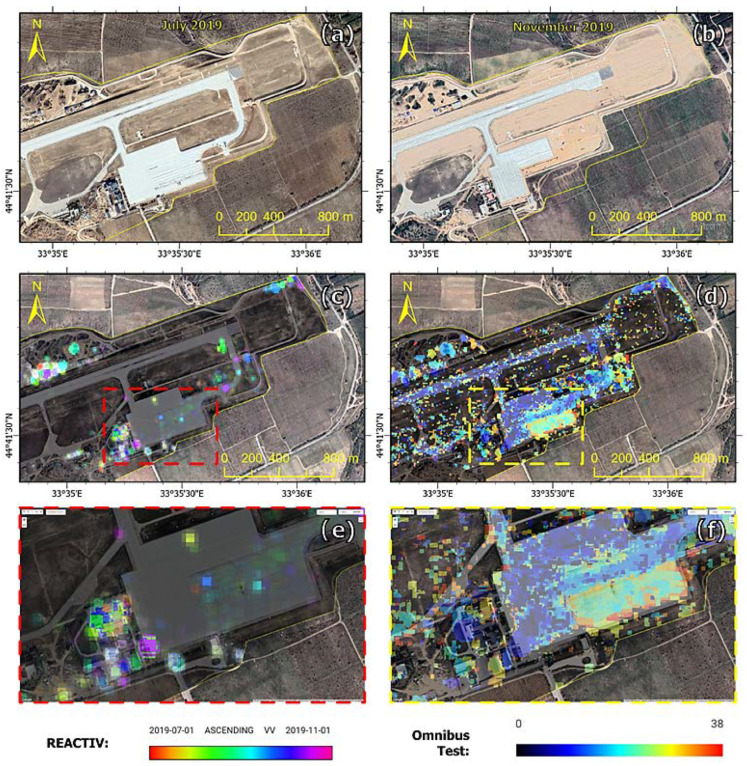
Comparison of changes detected in the Belbek Air Base (Area 2): (**a**) Google Earth Pro electro-optical image from July 2019, which detected changes, (**b**) Google Earth Pro electro-optical image from November 2019, which detected changes, (**c**) Our algorithm change detection, (**d**) Omnibus test change detection visualisation, (**e**) Zoom in to AoI (our algorithm), (**f**) Zoom in to AoI (Omnibus test).

The change detection method is completely data-driven and unsupervised: the physical reason for identified changes must be deduced from the context. The results of our methodology were compared with the method based on the Omnibus test, which functional principle is completely different and which uses dual polarisation (VV and VH) in the change detection. In the Omnibus method, the threshold criterion is the result of a hypothesis test for the homogeneity of all observed coherence matrices, which are assumed to obey the Wishart law. The criterion is then represented as the ratio of these matrices’ geometric mean and arithmetic mean temporal determinants. Validation of the results was carried out for two areas: POL storage in the Chkalovsk Air Base (Figure 17) and the modernised area of Belbek Air Base (Figure 18).

The first time series consisting of 42 Sentinel-1 images was analysed using REACTIV an algorithm, and its results are presented in Figure 17, which shows both the results of the original REACTIV version (Figure 17d) and our algorithm version (Figure 17c). There is a noticeable increase in interpretation potential. As a result of a visual comparison of images obtained from Google Earth Pro, a total of 17 changes in infrastructure were detected, of which, 14 changes are due to the construction of a new facility, and 3 others are due to the demolition of previously existing facilities. In check with respect to the validity or accuracy of the land cover changes captured by the REACTIV method in the time period from 1 March 2021 to 1 November 2021, it can be seen that the majority are “True positive”, i.e., really a building has been newly constructed (Table 2, Figure 17). A few are “False negative”, possibly because of too weak of a change in the land cover stored in the intensity image. The changes detected in the remaining area are possibly caused by ongoing groundworks. The time series from the second area consists of 38 Sentinel-1 images, and analyses of changes in the Belbek Air Base were carried out in the same way as in the case of the first area. The results of change detection (using two methods) in both areas are presented in Table 2.

The analyses concerned long-type events, for example, those related to the construction/deconstruction of a building, route, or runway. Both methods detected a similar number of changes. In the case of the REACTIV method, you notice a greater number of changes, also positive and false negative. This is due to the type of changes (surface changes—for example, the construction of a runway) and use single VV polarisation. The importance of chosen polarisation is also visible in build-up areas and areas that contain vehicles. On Sentinel-1 data, it appears that the VV polarisation highlights some objects much better than the VH polarisation. Overall, the performance of detection in the VV channel is better than for the VH polarisation. The Omnibus test method used dual (VV and VH) polarisation; this made it possible to detect surface changes. Both polarisations detect very few targets at once: the polarimetric information is, therefore, complementary. Merging the two polarisation channels improves performance. The disadvantage of the omnibus method is the occurring noise in the form of single pixels indicating a change.

## 4. Discussion

The proposed methodology enables fast, automated detection of changes in multitemporal series of radar imagery. Apart from detecting the change itself, the REACTIV method allows for expanding the results of change analysis by adding another dimension, i.e., determining the time of occurrence of change [44].

The application of gamma correction allowed to improve the radiometrics and increase the interpretation possibilities. However, the spatial resolution of the data used is a severe limitation, as it does not allow for detailed identification of the detected changes. This problem may be partly solved by applying fusion with high-resolution images in the visible spectrum, but access to these data is limited.

Due to very high relative electric permittivity (dielectric constant), metal objects strongly reflect microwaves. At an appropriate spatial resolution, metal objects such as aeroplanes are clearly visible in SAR images. On the other hand, the electric permittivity of natural elements (rocks, soil, and plants) or surfaces created by humans from non-metallic materials (asphalt or concrete) is usually significantly lower. Thanks to this phenomenon, aircraft located on stands at the airport are clearly distinguishable, which allows for their effective detection.

There is also a noticeable correlation between the obtained change detection results and the selected polarisation of radar data. The values of reflection intensity may differ significantly depending on the polarisation used. Polarised waves interact mainly with those structural elements of the scattering layer whose orientation is in line with the plane of polarisation. This is why vertically polarised waves (VV) interact better with spatial objects (those that stand out above the ground). The change of wave polarisation results from multiple scattering in the layer, and thus the recorded intensity of cross-polarised wave (VH) is lower than that of co-polarised wave (VV) [45].

However, the applied method has certain disadvantages. REACTIV does not visualise the direction, stability, or type of change in any way. REACTIV determines the scope of changes and the date of their occurrence based on the local maximum of the amplitude of the radar signal. Similar to the results by Agapiou [46], if regular changes are analysed, the hue component will be calculated based on the change of the highest maximum intensity value.

The proposed method of reducing this disadvantage is the use of a diagram of the distribution of changes in time (Figure 11 and Figure 15). The results confirmed the findings of other studies [47] that rapid decreases in intensity on the diagram might be interpreted as the disappearance of the object, while sudden increases represent its appearance.

Another way to work around the issue (especially when long-term changes are monitored) consists of dividing the analysed multitemporal series into stages (as it was completed in the analysis of changes in the POL storage area in Figure 8). This allows us to separate the periods characterised by different levels of intensity of changes.

An Earth Engine Compute Unit (EECU) is a technique for representing the amount of computing power available at any one time. As Google has a wide range of processing cores, architectures, and so on, EECUs are a good abstraction for discussing computational capacity. The number, kind, and architecture of machines working on a specific outcome can vary over time. We abstract all processing using EECUs since different physical cores can have different performance characteristics. Therefore, the assessment of the speed of the REACTIV method implemented in GEE is difficult. In [33], the authors compared the REACTIV and Omnibus methods. In summary, the two approaches outperform each other for detecting changes in large enough buildings. However, keep in mind that the full omnibus technique is time-consuming, whereas the other method is fast. The execution duration on the dataset, which consists of 64 dual-polarimetric pictures of (1133 × 3205) pixels, is 4 min for the REACTIV method and 50 min for the complete omnibus method on an Intel(R)Core(TM) i7-4710HQ CPU @ 2.50 GHz. However, it is noticeable that metode REACTIV is much more efficient compared to the Omnibus method. It is our experience that for Omnibus test algorithm very long image time series, typically more than 50 images, can lead to a stack overflow on the GEE servers. REACTIV based method can be used even for long-term time series, consisting of hundreds of images.

## 5. Conclusions

This study’s initial objective was to assess how well changes in long SAR images time series might be represented by a single image. This was accomplished using the new method based on REACTIV core, which makes it possible to visualise the changes that have been detected along with the date on which they occurred. However, it will not be possible to present all the information in a single three-channel image if multiple changes have taken place in the same area (such as an object that frequently appears and disappears). In this case, the algorithm visualises the colour that corresponds to the date of the most intense activity in the entire temporal series. Separating major changes from small changes is the biggest challenge in this kind of automatic method, which might be a very difficult problem and a suitable subject for some future research.

In order to answer the second research question, analysed the possibilities of the application of the algorithm in the multitemporal detection of changes in radar images and to evaluate the potential of applying the proposed methodology in the analysis of changes in the infrastructure and equipment of airports. The research works were based on the three main aspects of IMINT change detection: analysis of infrastructural changes, analysis of military activity, and Battle Damage Assessment (BDA), which were performed on military objects. The conducted research confirmed the effectiveness of the use of radar data in imagery intelligence. Observations with the use of SAR equipment may be successfully conducted at night or on cloudy days, even during rainfall. The essential element here is the continuity of observations, which is guaranteed because they may be conducted during each visit of the satellite above the area of interest, regardless of any adverse conditions mentioned above. Due to that, radar satellites are the perfect tools for detecting changes. Although algorithm makes it possible to see changes in picture stacks in a single image, there are a number of drawbacks from the standpoint of situational awareness. If there are many changes in the same location, the algorithm will merely link them to the date when the change was the greatest. The approach makes no attempt to determine the direction or stability of the change. The temporal resolution may also be an issue in huge image stacks because it might be challenging to tell one colour’s many shades apart from another.

Modern Earth observation systems provide enormous datasets. Archiving and storing them may require equipment and major expenditures. This results in the need to use computational clouds, which integrate the programming environment with disk resources. The Google Earth Engine platform that was used in the study provides an alternative for building its own IT infrastructure, with large disc space and the relevant computational power. It facilitates working on large datasets, which the multitemporal series of radar images doubtlessly are.

Constant monitoring of areas of interest and detecting the changes that occur there are the main tasks of satellite imagery intelligence, which enable to detection threats and update the state of knowledge about objects that may potentially pose such risks. The constantly growing dynamics of change in the security area results in the need to partly automate the intelligence processes. Automated systems usually perform analyses faster and more precisely than humans. The proposed methodology enables fast, automated detection of changes in multitemporal series of radar imagery. Apart from detecting the change itself, the method allowed for expanding the results of change analysis by adding another dimension, i.e., determining the time of occurrence of the change. The application of coloured visualisation of change enabled to broaden of the information capacity of the research results. Additionally, the generated maps of changes are characterised by an improved signal-to-noise ratio in the resultant images, which results from the reduction in speckle noise.

All the analyses presented in this study are based on open-source data. In spite of the limitations resulting from their spatial and temporal resolution, these data enable to satisfy the needs of imagery intelligence at least partly. It may be assumed that if data obtained from commercial or military systems with high temporal resolutions were used, the final results would become more up-to-date, and increasing the spatial resolution of the SAR input data would enable to detect changes in significantly smaller objects.

## Figures and Tables

**Table 1:** Specification of Sentinel-1 constellation [11].

Parameter	Specifications
Date of launch	Sentinel-1A—3 April 2014
	Sentinel-1B—25 April 2016
Orbit	Sun-synchronous polar orbit
Altitude	693 km
Sensor	SAR C band, band 5045 GHz radar
Pixel spacing (Acquisition mode: Interferometric Wide Swath, IW) Resolution	• 10 × 10 m (range × azimuth), for High Resolution class Level-1 GRD • 40 × 40 m (range × azimuth), for Medium Resolution class Level-1 GRD
Polarization	HH + HV or VV + VH (SM, IW, EW mode) HH lub VV (WV mode)
Repeat ground track	• 12 days (1 satellite) • 2–6 days (2 satellites)

**Table 2:** Overview of land cover changes detected by the REACTIV and Omnibus method in relation to the type of change.

Source		Constructed			Deconstructed			Sum
		True Positive	False Positive	False Negative	True Positive	False Positive	False Negative	
REACTIV	Area 1	13	2	3	-	-	3	59
	Area 2	26	3	6	2	-	1	
Omnibus	Area 1	10	-	6	1	1	2	58
	Area 2	32	1	2	2	-	1	

## Data Availability

Source codes are in the repository. URL: https://github.com/jakubslesinski/REACTIV_JS (accessed on 10 May 2023).

## References

[1] Cardillo R. (2018). Geospatial Intelligence Basic Doctrine. Collections. https://www.nga.mil/resources/GEOINT_Basic_Doctrine_Publication_10_.html.

[2] Clark R.M. (2007). Intelligence Analysis: A Target-Centric Approach.

[3] Munir A., Aved A., Blasch E. (2022). Situational Awareness: Techniques, Challenges, and Prospects. AI.

[4] İlsever M., Ünsalan C. (2012). Two-Dimensional Change Detection Methods.

[5] Zhao W. (2019). Multitemporal SAR Images Denoising and Change Detection: Applications to Sentinel-1 Data. Ph.D. Thesis.

[6] Li W., Ma P., Wang H., Fang C. (2023). SAR-TSCC: A Novel Approach for Long Time Series SAR Image Change Detection and Pattern Analysis. IEEE Trans. Geosci. Remote Sens..

[7] Hussain M., Chen D., Cheng A., Wei H., Stanley D. (2013). Change Detection from Remotely Sensed Images: From Pixel-Based to Object-Based Approaches. ISPRS J. Photogramm. Remote Sens..

[8] Mikkola J. (2018). Synthetic Aperture Radar Image Stacks in Support of Situational Awareness. Aalto University, Finland. http://urn.fi/URN:NBN:fi:aalto-201908254932.

[9] Jiang A., Dai J., Yu S., Zhang B., Xie Q., Zhang H. (2022). Unsupervised Change Detection around Subways Based on SAR Combined Difference Images. Remote Sens..

[10] Gao Y., Gao F., Dong J., Li H.-C. (2021). SAR Image Change Detection Based on Multiscale Capsule Network. IEEE Geosci. Remote Sens. Lett..

[11] Mastro P., Masiello G., Serio C., Pepe A. (2022). Change Detection Techniques with Synthetic Aperture Radar Images: Experiments with Random Forests and Sentinel-1 Observations. Remote Sens..

[12] Du Y., Zhong R., Li Q., Zhang F. (2022). TransUNet++SAR: Change Detection with Deep Learning about Architectural Ensemble in SAR Images. Remote Sens..

[13] Dekker R.J. (1998). Speckle Filtering in Satellite SAR Change Detection Imagery. Int. J. Remote Sens..

[14] Ban Y. (2016). Multitemporal Remote Sensing.

[15] Méndez Domínguez E., Meier E., Small D., Schaepman M.E., Bruzzone L., Henke D. (2018). A Multisquint Framework for Change Detection in High-Resolution Multitemporal SAR Images. IEEE Trans. Geosci. Remote Sens..

[16] Novak L.M., Blacknell D., Griffiths H. (2013). Advances in SAR Change Detection. Radar Automatic Target Recognition (ATR) and Non-Cooperative Target Recognition (NCTR).

[17] Hammer H., Kuny S., Thiele A. (2021). Enhancing Coherence Images for Coherent Change Detection: An Example on Vehicle Tracks in Airborne SAR Images. Remote Sens..

[18] Liu W., Yang J., Zhao J., Yang L. (2017). A Novel Method of Unsupervised Change Detection Using Multi-Temporal PolSAR Images. Remote Sens..

[19] Dai K., Li Z., Tomás R., Liu G., Yu B., Wang X., Cheng H., Chen J., Stockamp J. (2016). Monitoring Activity at the Daguangbao Mega-Landslide (China) Using Sentinel-1 TOPS Time Series Interferometry. Remote Sens. Environ..

[20] Gupta N., Ari S., Mishra A.K. (2021). A Novel Unsupervised Thresholding Technique for Landsat Image Change Detection. Proceedings of the Twelfth Indian Conference on Computer Vision, Graphics and Image Processing.

[21] Zhang X., Li Z., Hou B., Jiao L. Spectral Clustering Based Unsupervised Change Detection in SAR Images. Proceedings of the 2011 IEEE International Geoscience and Remote Sensing Symposium.

[22] Geng J., Wang H., Fan J., Ma X. Change Detection of SAR Images Based on Supervised Contractive Autoencoders and Fuzzy Clustering. Proceedings of the 2017 International Workshop on Remote Sensing with Intelligent Processing (RSIP).

[23] Cui B., Zhang Y., Yan L., Cai X. (2017). A sar intensity images change detection method based on fusion difference detector and statistical properties. ISPRS Ann. Photogramm. Remote Sens. Spat. Inf. Sci..

[24] Chen G., Zhao K., Powers R. (2014). Assessment of the Image Misregistration Effects on Object-Based Change Detection. ISPRS J. Photogramm. Remote Sens..

[25] Sofiane H., Ferdaous C. Comparison of Change Detection Indicators in SAR Images. Proceedings of the 8th European Conference on Synthetic Aperture Radar.

[26] Li L., Wang C., Zhang H., Zhang B., Wu F. (2019). Urban Building Change Detection in SAR Images Using Combined Differential Image and Residual U-Net Network. Remote Sens..

[27] Almeida-Filho R., Rosenqvist A., Shimabukuro Y.E., Silva-Gomez R. (2007). Detecting Deforestation with Multitemporal L-band SAR Imagery: A Case Study in Western Brazilian Amazônia. Int. J. Remote Sens..

[28] Clement M.A., Kilsby C.G., Moore P. (2018). Multi-Temporal Synthetic Aperture Radar Flood Mapping Using Change Detection: Multi-Temporal SAR Flood Mapping Using Change Detection. J. Flood Risk Manag..

[29] Datta U., Notarnicola C., Bovenga F., Bruzzone L., Bovolo F., Benediktsson J.A., Santi E., Pierdicca N. (2020). Infrastructure Monitoring Using SAR and Multispectral Multitemporal Images. Proceedings of the Image and Signal Processing for Remote Sensing XXVI.

[30] Pang L., Zhang F., Li L., Huang Q., Jiao Y., Shao Y. Assessing Buildings Damage from Multi-Temporal Sar Images Fusion Using Semantic Change Detection. Proceedings of the IGARSS 2022—2022 IEEE International Geoscience and Remote Sensing Symposium.

[31] Canty M.J., Nielsen A.A., Conradsen K., Skriver H. (2019). Statistical Analysis of Changes in Sentinel-1 Time Series on the Google Earth Engine. Remote Sens..

[32] Conradsen K., Nielsen A.A., Skriver H. (2016). Determining the Points of Change in Time Series of Polarimetric SAR Data. IEEE Trans. Geosci. Remote Sens..

[33] Colin Koeniguer E., Nicolas J.-M. (2020). Change Detection Based on the Coefficient of Variation in SAR Time-Series of Urban Areas. Remote Sens..

[34] Yuan J., Lv X., Dou F., Yao J. (2019). Change Analysis in Urban Areas Based on Statistical Features and Temporal Clustering Using TerraSAR-X Time-Series Images. Remote Sens..

[35] Inglada J., Mercier G. (2007). A New Statistical Similarity Measure for Change Detection in Multitemporal SAR Images and Its Extension to Multiscale Change Analysis. IEEE Trans. Geosci. Remote Sens..

[36] Kaplan A., Haenlein M. (2019). Siri, Siri, in My Hand: Who’s the Fairest in the Land? On the Interpretations, Illustrations, and Implications of Artificial Intelligence. Bus. Horiz..

[37] Fang B., Pan L., Kou R. (2019). Dual Learning-Based Siamese Framework for Change Detection Using Bi-Temporal VHR Optical Remote Sensing Images. Remote Sens..

[38] Shi W., Zhang M., Zhang R., Chen S., Zhan Z. (2020). Change Detection Based on Artificial Intelligence: State-of-the-Art and Challenges. Remote Sens..

[39] Koeniguer E., Nicolas J.M., Pinel-Puyssegur B., Lagrange J.M., Janez F. (2018). Visualisation des changements sur séries temporelles radar: Méthode REACTIV évaluée à l’échelle mondiale sous Google Earth Engine. Rev. Fr. Photogrammétrie Télédétection.

[40] Martino T.D. (2022). REACTIV—Implementation for Sentinel Hub Custom Scripts Platform. Sentinel Hub Blog. https://medium.com/sentinel-hub/reactiv-implementation-for-sentinel-hub-custom-scripts-platform-10aa65fd9c26.

[41] Kumar L., Mutanga O. (2018). Google Earth Engine Applications Since Inception: Usage, Trends, and Potential. Remote Sens..

[42] AWACS Aircraft on the Modern Battlefield [ANALYSIS]. https://defence24.pl/sily-zbrojne/samoloty-awacs-na-wspolczesnym-polu-walki-analiza.

[43] Institute Experts Provide Analysis on Iranian Attacks on U.S. Bases in Iraq|Middlebury Institute of International Studies at Monterey. https://www.middlebury.edu/institute/news/institute-experts-provide-analysis-iranian-attacks-us-bases-iraq.

[44] Gruenhagen L., Juergens C. (2022). Multitemporal Change Detection Analysis in an Urbanized Environment Based upon Sentinel-1 Data. Remote Sens..

[45] López-Martínez C., Pottier E., Hajnsek I., Desnos Y.-L. (2021). Basic principles of SAR polarimetry. Polarimetric Synthetic Aperture Radar. Principles and Application.

[46] Agapiou A. (2021). Multi-Temporal Change Detection Analysis of Vertical Sprawl over Limassol City Centre and Amathus Archaeological Site in Cyprus during 2015–2020 Using the Sentinel-1 Sensor and the Google Earth Engine Platform. Sensors.

[47] Manzoni M., Monti-Guarnieri A., Molinari M.E. (2021). Joint exploitation of spaceborne SAR images and GIS techniques for urban coherent change detection. Remote Sens. Environ..

